# Supplementary material for: In Situ Simultaneous Analysis of Nitrogen and Phosphorus Migration in Urban Black Odorous Runoff
Source: Int J Environ Res Public Health. 2022 Oct 14;19(20):13240. doi: 10.3390/ijerph192013240 (PMC9603257; doi:10.3390/ijerph192013240)
Supplement: Supplementary file 1 [file ijerph-19-13240-s001.zip › ijerph-1946921-supplementary.pdf]

Supplementary Information for

In-situ simultaneous analysis of nitrogen and phosphorus migration in urban black-odorous runoff

Figure S1. Site condition of the sampling sites.

Figure S2. Beta diversity analysis of PCoA showing the differences separate of microbial community composition at the different sampling sites.

Figure S3. Heat map for predictive analysis of bacterial function.

Figure S4. TOC concentration of sampling sites sediment.

Table S1. The longitudes and latitudes of the sampling sites.

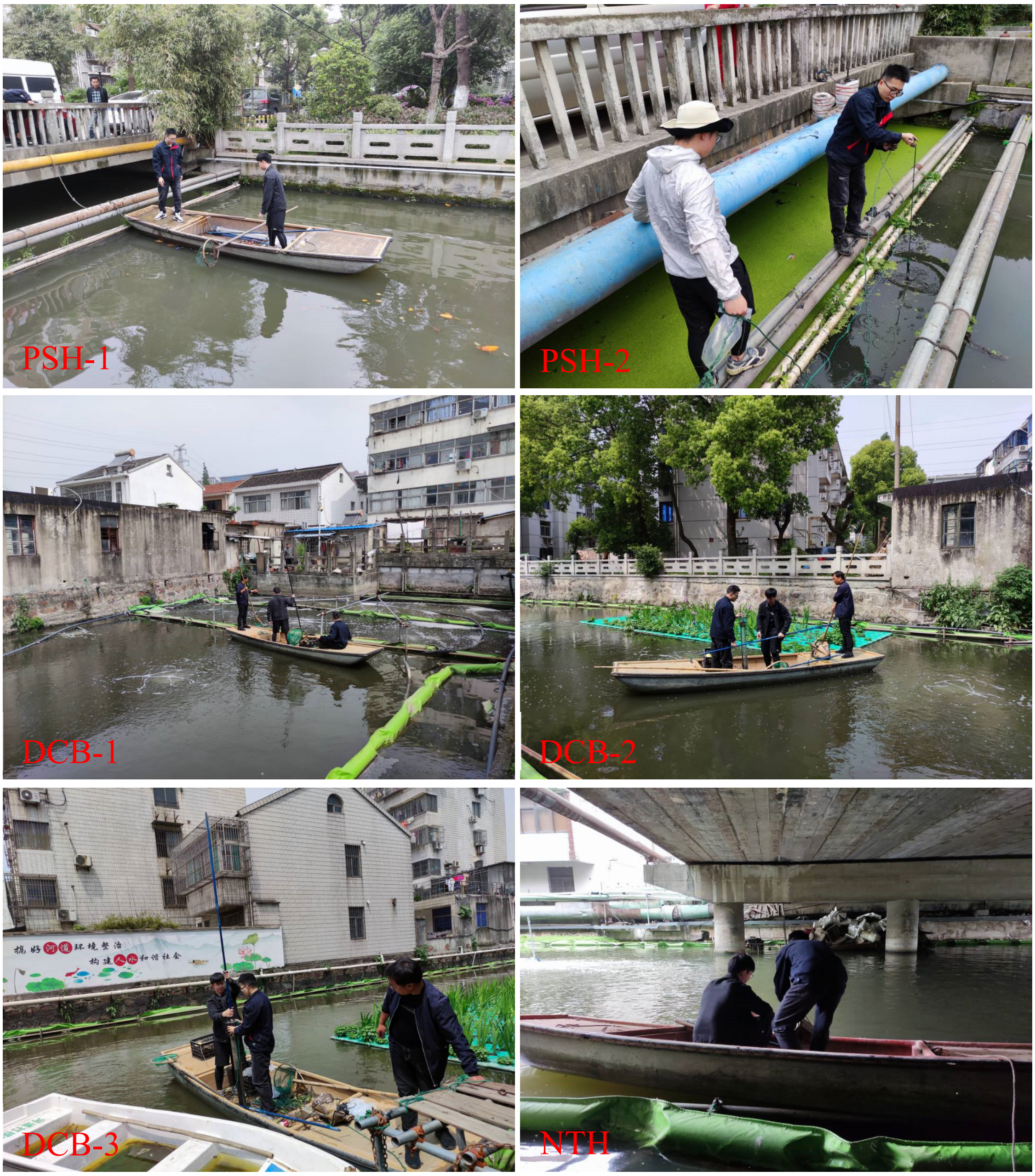

Figure S1. Site condition of the sampling sites.

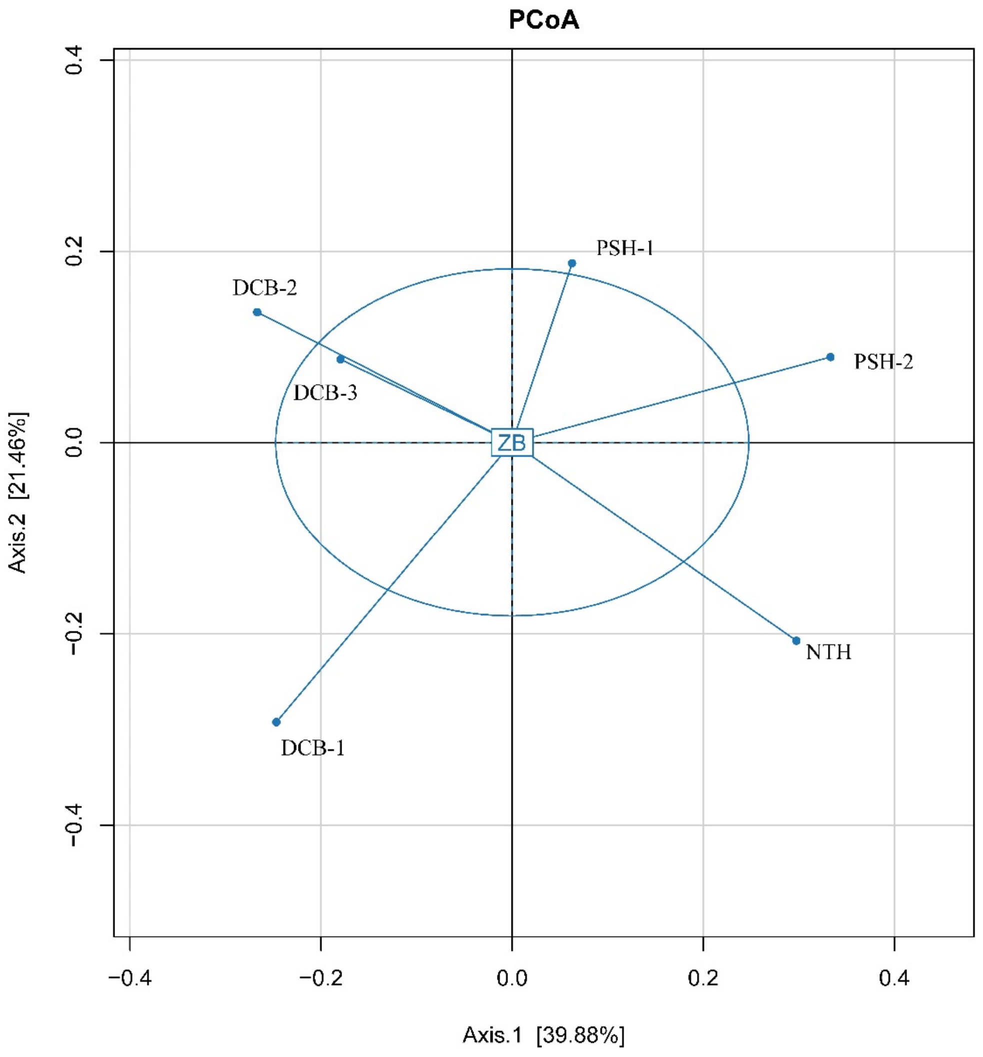

Figure S2. Beta diversity analysis of PCoA showing the differences separate of microbial community composition at the different sampling sites.

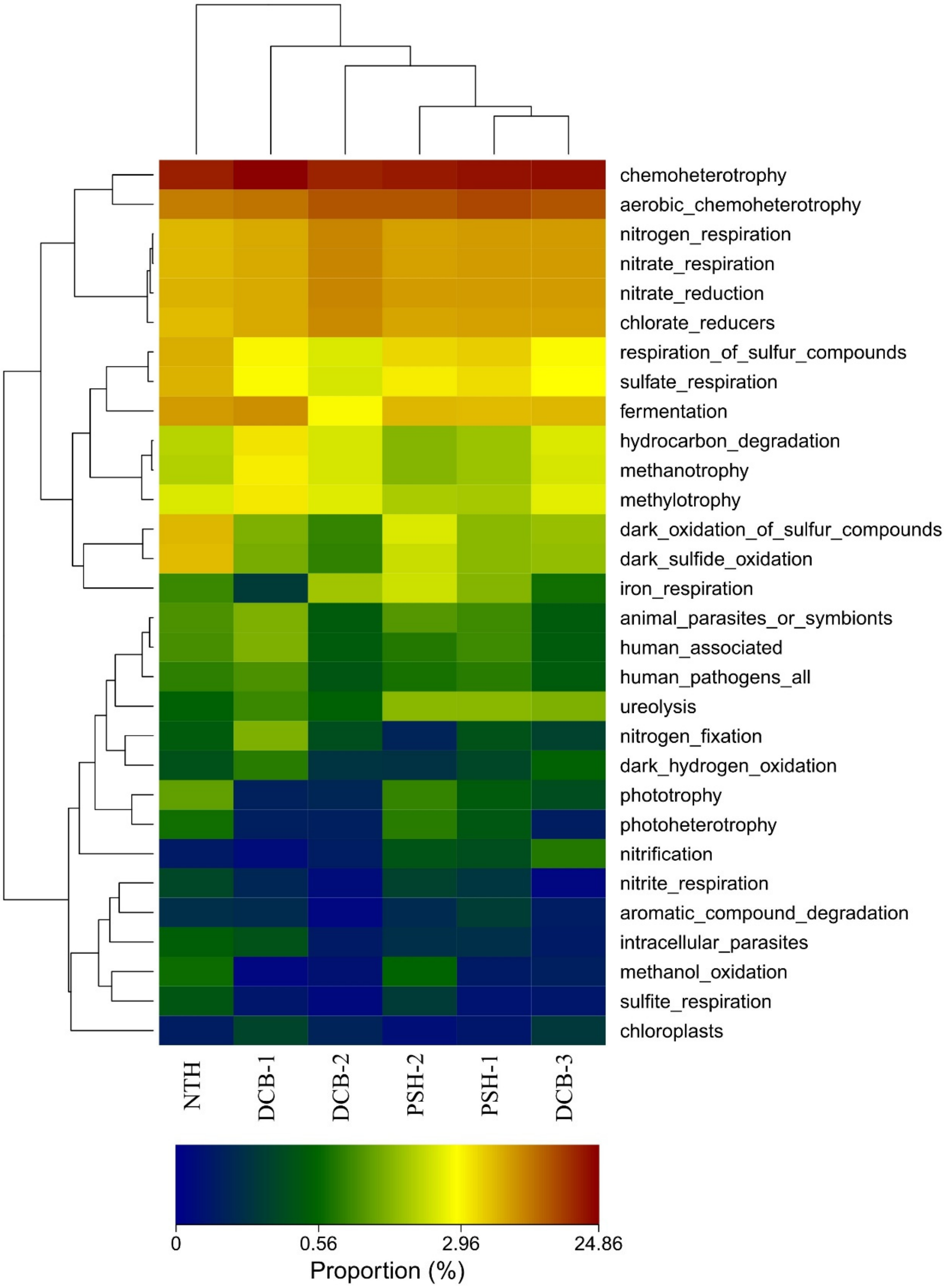

Figure S3. Heat map for predictive analysis of bacterial function.

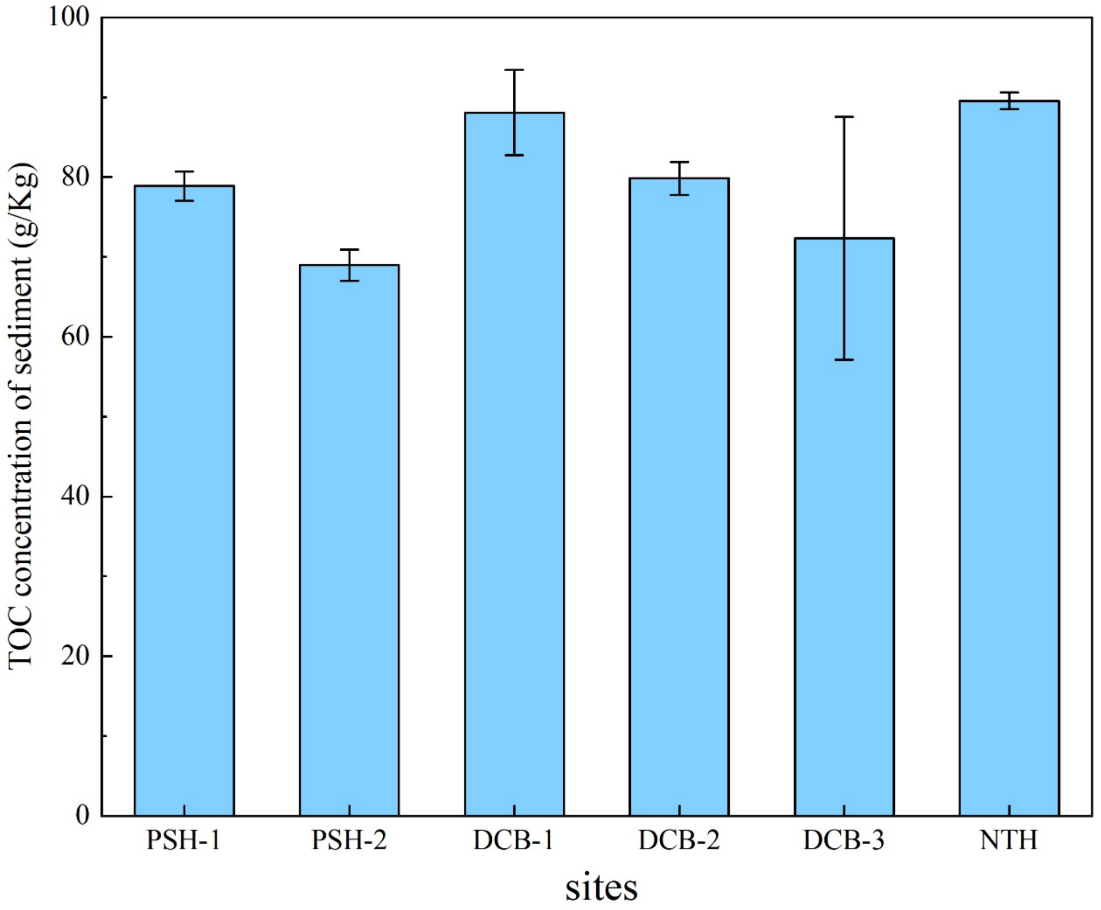

Figure S4. TOC concentration of sampling sites sediment.

Table S1. The longitudes and latitudes of the sampling sites.

Sampling sites	Longitudes (E)	Latitudes (N)
PSH-1	120.289876	31.600702
PSH-2	120.290542	31.601907
DCB-1	120.273155	31.558867
DCB-2	120.272997	31.557593
DCB-3	120.272813	31.556555
NTH	120.279340	31.593253
